# The Contribution of Human Herpes Viruses to γδ T Cell Mobilisation in Co-Infections

**DOI:** 10.3390/v13122372

**Published:** 2021-11-26

**Authors:** Fanny Martini, Eric Champagne

**Affiliations:** Infinity, Université Toulouse, CNRS, INSERM, UPS, CEDEX 03, 31024 Toulouse, France; fanny.martini@inserm.fr

**Keywords:** gamma delta T cells, lymphocytes, herpes virus, CMV, HHV, co-infections

## Abstract

γδ T cells are activated in viral, bacterial and parasitic infections. Among viruses that promote γδ T cell mobilisation in humans, herpes viruses (HHVs) occupy a particular place since they infect the majority of the human population and persist indefinitely in the organism in a latent state. Thus, other infections should, in most instances, be considered co-infections, and the reactivation of HHV is a serious confounding factor in attributing γδ T cell alterations to a particular pathogen in human diseases. We review here the literature data on γδ T cell mobilisation in HHV infections and co-infections, and discuss the possible contribution of HHVs to γδ alterations observed in various infectious settings. As multiple infections seemingly mobilise overlapping γδ subsets, we also address the concept of possible cross-protection.

## 1. Introduction

Viruses of the human herpes viridae (HHV) group have been co-existing with humans for millions of years, and existed in primates before the split of hominids and chimpanzees [1]. Eight species infect humans [2,3]: herpes simplex 1 (HSV-1/HHV-1), herpes simplex 2 (HSV-2/HHV-2), varicella–zoster virus (VZV/HHV-3), Epstein–Barr virus (EBV/HHV-4), human cytomegalovirus (CMV/HHV-5), herpes virus 6A and 6B (HHV-6A/B), herpes virus 7 (HHV-7) and Kaposi’s sarcoma virus (KSV/HHV-8). They are enveloped viruses with a large (125–235 kb) double-stranded DNA genome [4,5]. They perform a lytic cycle in epithelial cells which allows viral production, transmission and finally survival of the species. Immune responses normally control this infection, but HHVs share the capacity to persist indefinitely in the organism in a latent form with no or very limited viral replication. They can reactivate after the triggering of latently infected cells, leading to viral production and dissemination. An overt symptomatic reactivation remains a rather rare event considering the high prevalence of herpes family virus carriage in the population. With the exceptions of HSV-2 and KSV, HHV infections are ubiquitous. Most individuals worldwide become infected during the first decade of life and carry at least one HHV asymptomatically. Viruses of this family can thus be considered as part of the normal microbiome as well as the mucosal or cutaneous bacterial flora. The long-term co-evolution of these viruses with the hominid immune system allows the suggestion that some symbiotic relationship may have been established to be postulated, with an efficient immune control of excessive viral dissemination and, on the viral side, the development of diverse immune escape mechanisms [6,7]. The consequence of HHV carriage on the immune system is probably only partially estimated. T cell responses against CMV can mobilise a large part of T cells present in the elderly, and CMV in particular is known to shape the memory T cell compartment [8].

This review focusses on a particular subset of non-conventional T cells which use γ and δ clonotypic TCR chains in association with CD3 components to form their TCR. γδ T cell subsets are mobilised in acute infections by several HHVs, with sometimes-major expansions. They are at the interface between innate and adaptive immunity, and share with NKT or MAIT cells a limited TCR diversity due to their usage of a small set of TCR-variable genes. This restricted diversity reflects the recognition of relatively frequent ligands for which antigen-specific clones are present at a relatively high frequency. This allows them to perform their function after limited clonal expansion. This, added to the faculty of some of them to acquire an effector potential prior to antigenic challenge, allows them to respond quickly to infections. However, some subsets show adaptive-like behaviour, with massive clonal expansions and functional plasticity [9,10,11,12]. They also share with NK cells the expression of NK receptors and the capacity to be activated by TCR-independent signals and cytokines [9,10]. Subpopulations expressing preferential Vγ/Vδ combinations show preferential tissue locations in blood, lymphoid organs or specific tissues. Changes in the distribution and frequencies of γδ subsets are observed in viral, bacterial and parasitic infections, as well as in cancers. γδ T cells during infections can be directly cytotoxic on infected cells, inhibit pathogen replication or act as regulators of other actors of the immune response [9,10].

Although γδT cell subsets share common properties in different mammal species, they have diverged significantly. Differences between human and murine systems are reported for thymic differentiation processes as well as major γδ T cell subsets residing in blood and tissues [10]. Similarly, HHVs are strictly human with the exception of HSV1/2, which can infect mice. Homologs for CMV (MCMV), EBV/KSV (γ-herpes virus-68, MHV68) and HHV-6A/B (murine roseolovirus) exist, but they recapitulate only partially the characteristics of human infections [6,13,14,15]. Nevertheless, our knowledge of the impact of HHVs on the evolution and specification of γδ T cells remains limited.

Alterations of γδ subsets have been reported in the context of HHV infections and HHV-unrelated viral infections, including HIV, HCV, HEV, HBV, influenza, myxoviruses, flaviviruses and the vaccinia virus [16,17,18]. However, given the frequency of CMV, EBV and HHV-6 carriage in the general population, other viral infections in adults can generally be considered co-infections. The possibility of the concomitant reactivation of latent viruses constitutes a possible confounding factor for attributing γδ alterations to individual viruses. In this paper, we aimed to synthesise the available data relative to the contribution of HHVs to γδ T cell mobilisation and alterations during viral, bacterial or parasitic co-infections. We question the possible beneficial or detrimental effects of latent viral carriage on the immune control of co-infections through cross-immunity and shaping of the γδ T cell repertoire. Although BK, JC and Torque Teno viruses and adenoviruses (BKV, JCV, TTV and AdV) can establish latency and reactivate in conditions similar to HHVs, infections/co-infections with these viruses are not treated in our review, since data relative to γδ T cells in this context are virtually absent.

## 2. Frequency and Determinants of Latent Virus Reactivation

HHV latency is characterised by the persistence of the viral genome in an episomal form in the nucleus of infected cells. This is associated with no or very low viral replication, and with the expression of a restricted set of viral latency-associated genes. Viral reactivation from latently infected cells can be induced by factors which promote cell differentiation or activation as well as extrinsic environmental stress, such as hypoxia or inflammation [19]. Factors influencing reactivation can differ depending on the main site of latency. The reactivation of neurotropic HHV-1, -2 and -3 occurs in immunocompetent individuals following emotional or physical stress or local injury of tissues innervated by infected neurons. Stimuli which promote B cell activation, but also chemotherapy, UV or γ-irradiation preferentially promote the reactivation of EBV, which establishes latency mainly in B cells. CMV frequently reactivates in monocytes and their myeloid CD34^+^ precursors in multiple inflammatory contexts, in particular in situations where a strong allogeneic stimulation occurs, such as solid organ transplantation (SOT) or hematopoietic cell transplantation (HCT) [20,21]. Impairment of immune control, low T cell numbers and corticoid usage are, however, major factors of reactivation for most HHVs in the context of transplantation or the therapeutic control of inflammatory diseases [22,23]. In the absence of prophylaxis, and based on detectable viral DNAemia, the overt reactivation of CMV occurs in more than 50% of recipients of kidney allografts [24,25]. Reported frequencies for HSV and VSV are 53% and 4–12%, respectively [26,27]. After allo-HCT, the viruses most frequently reactivated were CMV, BKV and HHV-6 [28]. Multiple reactivations are frequent, and co-reactivations can occur simultaneously or successively [28,29]. The detection of multiple dsDNA viruses is associated with increased mortality and morbidity [30,31]. 

In the absence of obvious immunosuppression, reactivations of latent viruses are reported during sepsis with a frequency similar to that observed in HCT patients [32,33,34], during acute respiratory distress syndrome, during viral infections, non-tuberculous mycobacterial diseases [32,35] and non-infectious inflammatory contexts, such as atopic dermatitis [34] and rheumatic diseases [36]. 

## 3. Biological Features of γδ T Cells 

γδ T cells are the first T cells to appear in primary lymphoid organs during foetal development. In humans, six variable γ genes (TRGV) and seven variable δ genes (TRDV) are functional [37,38]. In the mouse system, subsets expressing specific Vγ/Vδ combinations differentiate sequentially in the thymus and make their home in different epithelial tissues, such as skin, intestine, tongue, lung or uterus. This is less clear in the human system, although two types of γδ T cells are clearly identified: a first wave of γδ cells expressing Vγ9 and Vδ2 first appear in the thymus, which is followed by a wave of cells expressing other V gene segments which predominate at delivery. Nevertheless, a thymic output of γδ T cells continues after birth [39]. Vγ9Vδ2 cells undergo a massive post-thymic expansion, and constitute the major subset found in blood (75–90% of γδ T cells) and lymph nodes in adults. In other tissues Vγ9^pos^Vδ2^pos^ T cells (Vγ9Vδ2) are usually present, but are outnumbered by other γδ cells expressing mainly Vδ1 and, less frequently, Vδ2 (Vγ9^neg^) and Vδ3, which pair with multiple Vγ genes with some tissue-dependent skewing [40]. 

With rare exceptions, the molecules identified as γδ TCR ligands are non-conventional MHC-related (EPCR, CD1b, CD1d, MR1, MICA, MICB and ULBP4) or MHC-unrelated self-proteins (annexin A2, ephrin A2, F1-ATPAse, hMSH2 and histidyl-tRNA synthase). Several of them are endogenous proteins which can be upregulated or expressed at the cell membrane under inflammatory or metabolic stress, or following malignant transformation. These ligands are recognised independently of associated peptides or lipids, and do not require the antigen processing machinery used for antigen presentation to αβTCRs. Few foreign antigens from pathogens have been reported to stimulate γδ TCRs (see [41] for a recent review). A particular case is represented by the semi-invariant human Vγ9Vδ2, most of which respond in a TCR-dependent manner to low-molecular-weight phosphorylated metabolites called phosphoantigens (PhAgs). These molecules can be endogenously produced (similar to isopentenyl pyrophosphate, IPP) or originate from bacteria or parasites (such as 1-Hydroxy-2-Methyl-2-buten-4-yl 4-diphosphate, HMBPP). Vγ9Vδ2 T cell activation by PhAgs requires cell contact with cells that are accumulating them. IPP overproduction may result from alteration of the mevalonate pathway of isoprenoid synthesis following viral infection [42,43], stress or malignant transformation [44,45], or experimentally after treatment with aminobisphosphonates (ABPs) [46]. HMBPP accumulation occurs upon infection with bacteria or parasites expressing the alternative 1-deoxy-D-xylulose-5-phosphate (DOXP) pathway of isoprenoid synthesis [47]. Vγ9Vδ2 T cell response to PhAg-expressing cells requires TCR-dependent co-recognition of isoforms of the butyrophilin family molecules BTN3A1 and BTN2A1, which can associate in multimers [48,49,50]. Most recent data indicate that the TCR does not bind PhAgs directly. Instead, PhAgs inside cells bind the intracellular B30.2 domain of BTN3A1, inducing a conformational change in the BTN3A1/BTN2A1 which is somehow sensed by the Vγ9Vδ2 TCR [50,51,52,53]. BTN recognition involves a major interaction of a framework region of the TCR γ-chain (HV4) with BTN2A1, a possible contribution of CDR2δ, and no apparent interaction of the TCR regions with the PhAgs or BTN3A1 [48,49,53]. Nevertheless, specific sequences in CDR3γ and CDR3δ determine PhAg responses: the length of CDR3γ is highly restricted to 14 ± 1 aa, and JγP (also called Jγ1.2) is almost invariably used. The length of this CDR3δ can be highly variable, but a hydrophobic residue is required in position five. The underlying mechanism is thus still incompletely understood. BTN complexes might serve to recruit unknown additional molecules or to position uncharacterised cell components for their interaction with the CDR regions of the Vγ9Vδ2 TCR [41,47,54]. Foetal Vγ9Vδ2 thymocytes are PhAg-responsive, suggesting that Vγ9Vδ2 lymphocytes are positively selected in the thymus for their response to PhAgs, possibly by endogenous metabolites such as IPP [38,39].

Other BTN isoforms have been shown to play a role in the tissue homing or maintenance of other γδ subsets in human and murine systems in a TCR-dependent manner. Thus, a general involvement of BTN isoforms in γδ T cell function and antigen recognition is suspected [55,56,57].

The comparison of the γδ TCR repertoire in cord blood and adult blood shows a progressive clonal focussing after birth, with the accumulation and predominance of a few clonotypes. This is thought to be driven by antigenic exposure, and CMV seems to be a major factor for the expansion of non-Vγ9Vδ2 clones (see below). These expansions display features of adaptive responses, and mainly involve private sequences [58,59]. Vγ9Vδ2 expansion observed after birth does not lead to substantial clonal focussing. Nevertheless, clonal or oligoclonal expansions in addition to public sequences in this subset are observable in some healthy individuals by examining Vδ2 CDR3 sequences and activated memory subsets [60,61,62,63,64,65,66,67,68,69]. Alterations of the repertoire occur with aging, influenced by persistent or recurrent pathogens, particularly viruses such as HHVs, which can persist throughout life and reactivate repeatedly [60,70].

Co-stimulatory, inhibitory and lymphokine receptors regulate γδ T cell functions (see [71] for review). Most γδ T cells also express variable combinations of activatory or inhibitory natural killer receptors, including natural cytotoxicity receptors (NCRs NKp30 and Np44), lectin-like receptors (NKG2A/C/D) and Ig-like receptors (activatory and inhibitory KIRs). Of special importance is NKG2D, a co-stimulatory receptor expressed on virtually all γδ T cells. NKG2D binds MHC-I-related ligands MICA, MICB and ULBP1-6 in humans, which are usually absent on the cell surface but are induced by stress conditions, such as tumoral transformation and viral infection [72,73]. Following infections by most HHVs, the upregulation of some NKG2D ligands is counter-balanced by viral products which interfere with the expression of selected NKG2D ligands known to limit NK cell cytotoxicity, resulting in an overall protection of infected cells [9,74]. Almost all human HHVs encode proteins or microRNAs which interfere with the expression of NKG2D ligands during lytic infection, thus limiting NK cell cytotoxicity [73] with rare exceptions [3,75], underlying the importance of NKG2D ligand interactions in host–virus equilibrium.

## 4. γδ and CMV/HHV-5

CMV infection usually occurs asymptomatically in the first months of life. When examined, 59.3% of Ugandan infants were found to be infected by CMV after one year [76], while the figure was 21.5% in Germany [77]. Seropositivity increases from 60% in European adults to 100% in African or Asian adults [78]. CMV replicates productively in multiple tissues, mainly fibroblasts and endothelial cells. Monocytes and CD34^+^ hematopoietic precursors constitute the main site of virus latency. Viral reactivation and replication from monocytes require cell stimulation and differentiation, and IFNγ and TNF are important factors which can promote viral production and dissemination to other tissues. This process is thought to occur at a low level in healthy individuals, and is tightly controlled by immune effectors, essentially CD8 cytotoxic αβ T cells and NK cells, whereas antibodies can limit dissemination to other tissues [4,7,79,80,81].

Expansions of Vδ2^neg^ γδ cells in the context of CMV infection were first reported after kidney transplantation (KTR), and subsequently in other contexts of immunosuppression [6,82,83,84,85,86,87]. In KTR, expanded cells in blood displayed CDR3 length restriction [88] and increased CD8 expression [87,88]. A higher level of γδ T cell expansion was associated with better control of infection [88,89,90]. A blood signature of CMV infection remained, characterised by elevated numbers of Vδ2^neg^ cells in blood, stable expression of a TEMRA late effector phenotype (CD45RA+, CD27− and CCR7−) and increased expression of activation markers (CD28, CD69, HLA-DR, perforin, granzyme B and CD16) and of co-inhibitory receptors PD-1, TIM-3 and LAG-3 [87,91].

The consequences of CMV infection on γδ T cells have also been examined after hematopoietic stem cell transplantation (HSCT) or cord blood transplantation (CBT). The reconstitution of the γδ T cell pool after HSCT or CBT occurs faster than the reconstitution of the αβ T cell compartment, and even faster in patients with reactivating CMV, whereas the reconstitution of Vδ2 cells is not changed [82]. Initial studies have examined the diversity of Vδ2^neg^ cells from HSCT patients by spectratyping CDR3δ [82,92], but did not detect clear differences between patients with reactivating CMV and controls [93]. However, recent investigations applying NGS technology have revealed massive proliferation of new clones which were essentially Vγ9^neg^Vδ2^neg^ following the reactivation of CMV after HSCT [58,94,95]. Clonal amplifications mainly involved private sequences, although the occurrence of public sequences was noticed [58,94]. Purified Vδ2^neg^ γδ cells from patients experiencing the reactivation of CMV, but not those from patients experiencing the reactivation of EBV, recognised specifically CMV-infected fibroblasts [82,93,96]. The roles of the TCR and NKG2D in this activation were not clear, and differed for cytotoxicity and IFNγ release [82]. In the Scheper et al. study [92], Vδ2^neg^ cells from patients having undergone HSCT/CBT for B cell chronic leukaemia (CLL) reacted in vitro against autologous B-CLL cells, and this reactivity required CD8αα in addition to Vδ1 TCR. Strikingly, however, transduction of the TCR from two clones highly reactive to CMV into αβ T cells conferred reactivity towards the leukemic cells, but not against CMV-infected fibroblasts. In another study, expanded Vδ1 did not react against autologous B-CLL cells [96]. These apparently conflicting observations suggest that different Vδ2^neg^ clones from patients could have been selected by alternative determinants, with a possible influence of the infected tissue. 

An imprint of CMV is also observable in healthy carriers, who usually display higher numbers of Vδ2^neg^ cells in blood when compared to CMV^neg^ individuals [82,92]. Asymptomatic immunocompetent blood donors who have been exposed to CMV have a higher percentage of TEMRA cells within the Vδ2^neg^ subset [70,82,91,97]. Further evidence for the mobilisation of Vδ2^neg^ cells by CMV comes from their expansion in neonates after in utero infection [98]: although clonal amplifications with essentially private sequences were observed, a public foetal Vγ8Vδ1 TCR containing germline-encoded CDR3 sequences was found amplified in all six tested CMV-infected newborns [98]. As these cells responded in vitro to CMV-infected targets and showed antiviral activity, they may participate in the control of CMV infection during foetal life [98].

An MHC-like protein, endothelial protein C receptor (EPCR), has been identified as a ligand for a Vγ4Vδ5 highly expanded clone representing 25% of blood T cells in a CMV-infected patient. The expression of this antigen does not seem to be modified by CMV infection [99]. Nevertheless, the TCR of this clone was later shown to bind the butyrophilin molecule BTNL3 in a Vγ5-HV4-dependent interaction, similar to that of the Vγ9 TCRs with BTN2A1, indicating a dual-modality interaction of this TCR with different ligands. Whether these different interactions contribute simultaneously or not to γδ T cell reactivity against CMV is currently unknown [100].

Chronic inflammation as well as aging are associated with increased oxidative stress, which promotes the reactivation of CMV; conversely, the reactivation of CMV promotes oxidative stress [101,102]. Recently, a Vγ9Vδ1 T cell clone expanded in a CMV-infected patient was found to recognise EphA2, an endogenous protein which is upregulated on multiple tumours and is a marker of metabolic dysregulation. EphA2 recognition involves the clonotypic regions of the TCR. This suggests that some Vδ1 clones expanded in CMV infection function to detect metabolic dysfunction [103]. Three non-Vγ9Vδ2 clones isolated from unrelated healthy donors, expressing different Vγ/Vδ TCR combinations, were found to recognise, in a TCR-dependent manner, annexin A2, an antigen upregulated on the surface cell lines and tumours following hypoxia or CMV infection [104].

Thus, the non-Vγ9Vδ2 γδ T cell compartment displays an adaptive behaviour and CMV appears to be one major driver of clonal amplifications, possibly by inducing the expression of multiple stress ligands, which are also expressed on tumours.

## 5. γδ and EBV/HHV-4

EBV seroprevalence is over 90% in the worldwide population [105,106,107,108]. In most instances primo-infection occurs in early childhood, and is asymptomatic due to an efficient immune control in which CD8 cytotoxic αβ T cells and NK cells play a major role [109,110,111]. When infection takes place later in life it causes a transient symptomatic oropharynx infection, infectious mononucleosis (IM), characterised by a massive CD8 αβ T cell expansion. EBV is an oncogenic virus responsible for lymphomas and epithelial cancers. This oncogenic potential has been reviewed elsewhere, and is not addressed in this review [112,113]. After the infection of B cells different sites of latency have been described, during which limited sets of latency-associated viral genes are expressed. Latency 0 (defined by viral genome persistence without transcription) and latency I (single expression of EBNA-1) states are found in cycling memory B cells and Burkitt’s lymphoma (BL); latency II (EBNA1^+^, LMP1^+^ and LMP2^+^) is found in germinal centres of EBV carriers, Hodgkin’s lymphoma and some in-vitro-generated EBV-infected lymphoblastoid cell lines (LCLs). Latency III (expression of multiple LMPs, EBNAs and mRNAs) is associated with EBV infection of naive B cells, lymphomas occurring in EBV-associated lymphoproliferative disease (EBV-PTLD) and most EBV-LCLs [114]. The virus infects primarily B cells, but also epithelial cells, and persists in a latent state mainly in resting memory B cells [115,116,117]. Occasional reactivations occur predominantly in mucosa-associated lymphoid tissue [118].

A specific reactivity of γδ T cells against some EBV-infected targets is substantiated by in vitro studies. γδ T cells expressing Vγ9-JP/Vδ2 are responsive to latency I EBV^+^ Burkitt’s lymphoma cell lines Daudi and Akata, whereas no response is observed against latency II or latency III EBV-LCLs [119,120]. Moreover, the responses to Akata and Daudi are dependent on BTN3 recognition and can be abrogated by downmodulating PhAg production with statins [46], or by blocking the TCR/CD3/BTN3 axis [50,119,120]. Thus, the reactivity against EBV latency I B cell lines relies at least in part on PhAg detection.

Healthy donors segregate into two populations according to the magnitude of their in vitro response to PhAgs and Akata [62,119,120]. This bimodality appeared to be independent of age, sex, CMV or EBV status. In one study, amplified cells seemed to also differ functionally, as Vγ9Vδ2 from high responders produced more IFNγ and MIP1β, and were more cytotoxic upon stimulation. This dimorphism arises after birth prior to EBV or CMV infection, and is not found in a neonatal thymus [62]. An unknown process acting at the level of γδ T cell development could be involved, leading to the predominance of Vγ9-JγP (PhAg-responsive) versus Vγ9-Jγ2 (non-responsive) rearrangements within the Vγ9/Vδ2 subset. Alternatively, early exposure to PhAgs after bacterial or parasitic infections might fashion this repertoire, including exposure to HHV-1/2, HHV-6, recurrent viral infections or vaccination (see below) [61,121].

At the acute phase of IM, patients have elevated numbers of CD8, CD4, NK, iNKT and γδ cells [119,122,123,124]. Vδ2^pos^ cells are amplified in blood, display an activated phenotype (DR^+^, CD38^+^) and persist for several months at the convalescent phase [125,126]. IM children were also found to segregate into two populations according to their proportion of Vδ2 T cells among PBMCs at the acute phase of infection, whereas all of them had similar NK responses. This bimodality was present in EBV-seropositive children of the same age who had presumably been recently infected asymptomatically, but was not found in healthy adults who perfectly controlled EBV infection. Thus, EBV was responsible for the persistence of increased numbers of Vδ2 T cells in seropositive healthy and primo-infected children. The proportion of high responders in symptomatic IM patients was lower than that in EBV^+^ healthy children who presumably encountered EBV asymptomatically, suggesting that high Vγ9Vδ2 responses in IM are linked to a reduced symptomatology [120].

Analyses of γδ T cells after HSCT also support the involvement of Vγ9Vδ2 cells in EBV responses. In a large cohort including 110 EBV^neg^ patients on an EBV-DNAemia basis and 22 patients with the post-HSCT reactivation of EBV [127], all patients showed a better reconstitution of the Vδ1 compartment compared to Vδ2, and had a predominant Vδ1 population 2–3 months after HSCT. This was attributed to the fact that almost all individuals also experienced the reactivation of CMV. The study also pointed out that patients with higher Vδ2 counts experienced a lower incidence of the reactivation of EBV. Phenotypic analysis of Vδ2 cells revealed expression of CD38 and HLA-DR on Vδ2^+^ cells selectively in EBV^pos^ patients. Thus, Vδ2 cells were activated following the reactivation of EBV, and may have participated in the control of EBV infection/reactivation. In contrast, another study linked an increased oligoclonality within the Vδ1 subset to the reactivation of EBV after allo-HSCT. However, the role of CMV was not excluded [128].

IM is generally resolutive. In rare cases, reported mainly in East Asia, it evolves on a chronic mode called chronic active EBV infection (CAEBV), with recurrent IM-like episodes. In this paediatric disorder, T and NK cells get infected by EBV in a context of high viral load, possibly after contacting infected B cells, and infiltrate multiple organs. These infected T/NK cells then apparently disappear to become undetectable after one year. Starting as a systemic inflammation, the disease can become malignant, leading to hemophagocytic lymphohistiocytosis (HLH) or lymphoma transformation characterised by monoclonal NK or T cell expansions [129]. Clonal expansions of EBV-infected Vγ9Vδ2 T cells have been observed in a form of the disease with a skin manifestation, hydroa vacciniform [130,131,132,133]. Thus, EBV infection of γδ T cells can occur, likely resulting from a cognate interaction between Vγ9Vδ2 cells and infected B cells. Through the recognition of EBV-infected B cells, Vγ9Vδ2 T cells may participate in the control of EBV-induced lymphomagenesis. This was suggested by an in vivo model of lymphomagenesis after transferring EBV-infected human cord blood cells to NSG mice and subsequently administrating ABP-activated Vγ9Vδ2 cells [134].

Another manifestation of acute EBV infection is post-transplantation lymphoproliferative disorder (PTLD), a syndrome linked to immunosuppression and associated with the reactivation of EBV. In PTLD, the reactivation of EBV in B cells induces an outgrowth of infected B lymphocytes. In more advanced stages this can lead to B cell transformation and lymphomas [135]. Cases of EBV-PTLD associated with an elevated level of γδ cells in blood and brain lesions have been reported [136,137]. Amplified cells belonged to the Vδ1 subset [137], and were reactive to autologous EBV-LCLs in vitro. Thus, the γδ cells that expand during EBV-PTLD closely resemble Vδ1 cells isolated after the stimulation of normal blood with EBV-LCLs. In these studies, activation was found to be dependent on cell contact, LFA1 and the TCR/CD3 complex. The putative activating ligand was of cellular origin rather than a viral structure, and was upregulated on activated B cells. LCL-responsive clones did not show predominant CDR3 sequences, and no evidence of oligoclonal expansion was found [138,139,140,141]. Remarkably, Vδ1 cells are also amplified in patients with acute B lymphocytic leukaemias [142,143], and in one study these cells were reported to lyse EBV^neg^ B-CLL cells. This suggests recognition of a cellular rather than viral determinant, but TCR-dependence of this activity was not reported [144]. A humanised murine model of EBV-lymphoproliferative disease has been used by administrating of EBV-LCL cells to B-cell-deficient Rag-/- mice. Strikingly, Vγ9Vδ2 cells transferred into these mice were able to prevent this experimental lymphoproliferative disease, in which transferred B cells were of a latency III type. In this work, Vγ9Vδ2 cells also killed EBV-LCLs in vitro. Nevertheless, ABP-preactivated Vγ9Vδ2 cells were used in vivo as well as in vitro, and their activity against EBV-LCLs was essentially mediated by NKG2D and Fas/FasL, rather than TCR-mediated [145]. 

Taken together, above studies reveal that EBV infection, symptomatic or not, can be detected by the Vδ2 and the Vδ1 subsets, which might contribute by recognising infected B cells at different stages of maturation/differentiation.

## 6. HSV1/2 Infection and Co-Infections

HSV1 and HSV2 are reported to infect, respectively, 66% and 11% of the worldwide population [146]. HSV1/2 infect mucosal tissues in human and mice, and establish latency in ganglionic neurones after retrograde axonal transport. Most cell types can, however, be infected productively through multiple receptors, including CD4+ αβ T cells [147]. In a murine model, γδ T cells accumulate in infected ganglia and can control lethal infection in αβ-deficient animals. HSV-specific γδ T cell clones have been isolated, and one was found to recognise a viral antigen in an unprocessed form and in a non-MHC-restricted way. Nevertheless, the γδ response in infected ganglia was polyclonal, and it was suggested that some γδ T cells recognise virally induced cellular determinants [148,149]. A protective role has been ascribed to IL-17-producing γδ cells in a corneal infection model, whereas γδ cells were ineffective in experimental cutaneous or vaginal infections [150,151], or were shown to increase pathology [152,153,154,155]. How these observations in mice can translate to human infections is not known. No systemic expansions of γδ T cells as those seen with CMV or EBV have been reported for HSV1/2 in humans. However, blood γδ cells from HSV-seropositive individuals expand in vitro in response to autologous HSV-infected PHA blasts [121,156]. In a study by Bukowski et al., expanded cells also recognised and killed Daudi BL and cells infected by the unrelated vaccinia virus, suggesting recognition of common virus-induced determinants. The reactivity was present in Vγ9Vδ2 cells and appeared MHC-unrestricted. Retrospectively, this suggests PhAg sensing. Interestingly, Maccario et al. showed that the cytotoxicity against HSV-1 cells involved mainly conventional CD8 cells in some individuals, whereas γδ cells were the main players in others, which is in line with the Vγ9Vδ2 population dimorphism described above for responses to EBV. Cell lines derived from lesion infiltrates of HSV-1-/HSV-2-associated genital herpes patients appeared enriched in Vγ9Vδ2 T cells [157]. Intriguingly, whereas EBV-LCLs are usually not targeted by Vγ9Vδ2 cells, in the Maccario et al. study, Vγ9Vδ2 cells killed autologous EBV-LCLs superinfected with HSV-1, suggesting a possible interaction. This was not observed in the other report [157], a discrepancy which might come from a variable efficiency of LCLs to properly express stimulatory determinants, such as BTNs, after infection.

## 7. γδ and HHV-6/7

It is estimated that 60 to 70% of the population harbour HHV-6 and HHV-7 in their salivary gland [158]. Both viruses target primarily CD4 αβ T cells, although their entry into cells is mediated by different receptors [159]. However, they can infect lymphoid, myeloid and epithelial cells. γδ T cell expansions have not been reported in response to HHV-6/7 infection. In vitro, HHV-6 superinfection can reactivate EBV in Akata and P3HR-1 latently infected cells lines, and upregulates Zebra EBV early antigen in EBV-non-producer Burkitt lymphoma Raji [160]. In addition, HHV-6 can productively infect CD8 T αβ cells, NK cells and γδ T cells, leading to CD4 expression on γδ cells and rendering them susceptible to HIV infection [161,162,163,164], a mechanism which may contribute to Vδ2 depletion in AIDS (see below). 

## 8. γδ and KSV/HHV-8

KSV is not ubiquitous. It’s prevalence in some Eastern or African areas may reach 50%; however, in Western countries, the prevalence varies from 3 to 23% and is tightly linked to HIV infection, due to frequent co-transmission [165,166]. Similar to EBV, KSV infects primarily cells of the B lymphocyte lineage, where it can establish latency. It also shares with EBV an oncogenic potential, leading, in particular, to primary effusion lymphoma [165,166,167]. A role for γδ T cells in HHV-8 infections has been reported in one study [168], where patients selected for shedding HHV-8 in saliva (n = 7) were compared to seronegative controls. An increase in peripheral Vδ1 cells was constantly observed, with a dominance of Vδ1 over Vδ2 in all HHV-8 infections. Expanded Vδ1 cells had a terminally differentiated phenotype characterised by CD57 expression. Vδ1-TCR CDR3 sequences analysis of seropositive individuals detected oligoclonal amplifications which were patient-specific, and thus comparable with the Vδ1 amplifications observed in the context of the reactivation of CMV. These cells proliferated preferentially in vitro after stimulation of PBMC with HHV-8 viral particles and IL-2, but also with several viral proteins, although in a weaker fashion. T cell lines derived from these individuals increased their lymphokine production when stimulated by the HHV-8-infected cells and several individual recombinant HHV-8 proteins. This response was diminished by the presence of anti-CD3 antibodies. However, activation was measured in the presence of PBMCs or bystander B cells (BJABs), which could have captured viral particles and/or produced cytokines, thus leaving open the possibility that Vδ1 recognised virus-induced determinants.

## 9. VZV/HSV-3 Infection

In temperate countries, 90% of the population is infected with VZV by adolescence, while inhabitants of tropical countries are infected later in life [169]. The reactivation of CMV in heart transplant recipients is a risk factor for the reactivation of VZV. Interestingly, VZV upregulates the NKG2D ligand MICA on the surface of infected cells, possibly stimulating NK cells (reviewed in [170]). Recurrent aphthous ulcers are mucosal lesions which have been associated with VZV infections and reactivation, even though other infections could possibly be involved. Increased γδ cells were found in blood during their exacerbation periods [171,172]. An infiltrate of γδ T cells, but also conventional T cells, was found in lesions by immunocytochemistry. The γδ subset putatively involved is unknown [173].

## 10. HHV Co-Infections 

In the context of immune suppression and transplantation, the infection/reactivation of multiple HHVs is frequent, CMV-HHV-6 being the most frequent co-infection [29,174]. In solid organ transplantation or HSCT, the reactivation of HHV-6 is frequently detected earlier than CMV and is a predictive factor for the reactivation of CMV [175,176,177,178,179]. After cord blood transplantation, HHV-6 usually reactivates after EBV [180]. HHV-6/7 infections increase the symptomatology associated with CMV, and HHV-7 increases the frequency of CMV disease [160]. The frequency of EBV-CMV co-infection post-transplantation varies between clinical studies, from 2.6 to 32.7% [31,179,181]. In the clinic, primary EBV infection does not reactivate CMV, but primary CMV infection may induce the reactivation of EBV [182,183,184]. In vitro, B cells infected by EBV are more susceptible to HHV-6 infection, and superinfection by HHV-6 can reactivate EBV [160]. The increased syncytia formation due to the formation of complexes between the glycoproteins gH (CMV) and gL (HHV-6) may play a role in the increased pathogenicity of co-infection by increasing viral spread [185]. TNF induced during HHV-6 infection was proposed to mediate the reactivation of CMV in solid organ transplantation [186]. In vitro, CMV can reactivate EBV in BJAB and P3HR-1 Burkitt’s lymphoma cell lines [187].

In spite of these multiple interactions, how co-infections with multiple HHVs affect γδ compartments is not known. Analysis of γδ T cells’ cross-reactivity against different HHVs is reported in few instances [88,168,188]. In one case, clones reacting against CMV did not proliferate in response to fibroblasts infected by HSV or VZV [88,188]. Nevertheless, in view of findings reported above, CMV-reactive and HSV-reactive γδ cells are recruited among different γδ subsets, and the targeting of VZV by any γδ subset is still uncertain. Interestingly, HHV-8 infection could be targeted by Vδ1 cells, which were found to be unreactive against CMV [168]. It is possible that fundamentally different strategies are used for the recognition of these two HHVs by γδ cells. Indeed, direct recognition of viral particles is suggested in the case of HHV-8, whereas recognition of endogenous proteins is currently a preferred model in the case of Vδ1 reactivity to CMV. Another possibility is that the absence of cross-reactivity is due to co-recognition of the cell lineage/tissue origin, which is different for CMV and KSV if one considers the major site of latency. BTN isoforms and multimers are good candidate molecules which could possibly confer tissue specificity after co-recognition [10,189]. 

The possible cross-reactivity between HHV-1/2-reactive and EBV-reactive γδ is another open question. Vδ2 cells are possibly targeted in both contexts, and PhAg-reactive cells are involved in both cases. To our knowledge, an analysis of possible cross-reactivity of Vδ2 cells against cells infected by these viruses is not reported.

## 11. γδ in HIV Infections

The effects of HIV on γδ have been reviewed recently [190,191]. Along with CD4 depletion, numbers of Vγ9Vδ2 in blood are severely decreased at the early phase of HIV infection, leading to an inverted Vδ2^pos^/Vδ2^neg^ ratio. This may precede inversion of the CD4/CD8 ratio [192]. Importantly, Vδ2 depletion correlates with HIV load and CD4 depletion [193]: it is reversed by antiretroviral therapy (ART), at least partially, and Vδ2 cells are lost again in patients following the interruption of treatment [194,195]. The cells responsive to PhAgs (characterised by Vγ9-JP usage and characteristic CDR3 sequences) are preferentially depleted, and the remaining Vγ9Vδ2 cells have been found to be hypo-responsive to PhAgs. Interestingly, a rapid decline in Vγ9Vδ2 T cells is also observed following SIV infection of macaques, and is preceded by a transient expansion, suggesting activation. In line with this, a specific in vitro activation of Vγ9Vδ2 cell lines derived from healthy subjects against HIV-infected targets has been observed [16], and PhAg-activated cell lines can kill HIV-infected targets [196]. Vδ2 are preserved in blood in HIV elite controllers (ECs, also called natural viral suppressors) [197]. However, TCRγ repertoire analysis revealed that they have a lower proportion of Vγ9-JP rearrangements corresponding to PhAg-responsive cells [198]. It is not clear, however, if this feature is due to HIV carriage or if most ECs belong to the subtype of individuals with naturally weak PhAg responses mentioned above [62,119,120]. Although the cause of Vδ2 cell depletion in progressors is not clear, several mechanisms have been identified which can contribute to it (see [191] for a review). As mentioned above, HHV-6 infection can promote HIV infection of Vδ2 cells [161,162,163,164]. 

Paralleling Vδ2 depletion, a persistent increase in Vδ1 T cells occurs in the blood of HIV-viraemic patients, and may start prior to seroconversion [199,200]. This is also observed in SIV-infected macaques [201]. Repertoire analysis of Vδ2^neg^ cells in HIV patients did not show clear differences with healthy controls, arguing for an unbiased polyclonal or oligoclonal amplification [202,203]. Indirect observations suggest that Vδ1 amplification is not a direct effect of HIV infection: Vδ1 amplification does not correlate with HIV load; in contrast to Vδ2 depletion, it occurs in ECs, although to a lesser extent, and is not reversed by ART [197,204]; and high Vδ2^neg^ counts in some ECs do not correlate with the presence of HIV in blood, but parallel increased HIV replication in gut [204].

Several factors possibly contributing to Vδ1 expansion have been identified. (a) A stimulation by opportunistic pathogens is possible, as the highest Vδ1 counts were observed in the context of co-infection by Candida albicans [205]; in vitro, Vδ1 expanded from HIV patients responded to Candida albicans [206] and Mycobacterium avium [207]. (b) An increase in Vδ1 is observed in the gut and reproductive tract where HIV replication occurs [194,208,209], also supported by observations in the macaque model [201,210]; the increase in blood Vδ1 cells may thus result from a mobilisation from the gut or tissues, possibly favoured by an alteration of the epithelial homeostasis [204,206,208]. A systemic release of HIV-tat peptide could also interfere with Vδ1 chemotaxis towards tissues [211]. 

## 12. HIV-HHV Co-Infections

Most HIV patients carry HHVs subclinically. One study followed viral shedding in semen by HIV-infected men who have sex with men (MSM), in a 48-week follow-up. HHV secretion was found in 94% of cases, with the following percentages for different viruses: 85% (CMV), 81%(EBV), 35% (HHV-7), 29% (HHV-6) and 26% (HSVs) [212]. CMV was found in vaginal secretion in 78% of HIV-infected women in Uganda in a similar time window [213]. Considering the frequency of HHV carriage in the HIV-infected population, it is extremely difficult to dissociate the effects of HIV from those of CMV. In murine LCMV infection, viral reactivation occurs mainly in tissues, and the reactivation of HHV is thus presumably underestimated in human studies where viral infection/reactivation is assessed in blood [214]. Individuals carrying HSV-1/2 display an increased risk of acquiring HIV, possibly because HSV infection induces epithelial lesions favouring HIV entry [215,216,217]. The prominent effect of CMV on NK cells in the course of HIV infections is well-documented and provides evidence for HIV–CMV interaction. CMV infection results in a dramatic change of the expression of NKG2A/C HLA-E-binding receptors on NK cells. NKG2A is an inhibitory receptor expressed on early differentiated NK cells, whereas NKG2C is activatory and expressed on terminally differentiated memory-like NK cells [167]. During CMV infection NKG2C^+^ NK cells expand while NKG2A^+^ NK cells are lost in transplant patients, but also in healthy CMV^+^ children [218,219,220]. A similar change was observed in HIV patients, possibly driven by the reactivation of CMV as it does not correlate with HIV virus load and persists on ART [221,222,223]. Moreover, when comparing HIV patients and controls, the difference in NKG2A/C frequencies disappears when patients are stratified for CMV [224]. In comparison, EBV and HSV-2 do not promote NKG2C^+^ NK cell expansion [225,226]. NKG2A/C receptors are also expressed on Vδ1 and Vδ2 cells [227]. In the blood of HIV-viraemic patients the proportion of NKG2A^+^ Vδ1 cells decreases, while NKG2C^+^ Vδ1 cells increase in number and display higher cytolytic activity on HLA-E-expressing targets [208,214,228]. Data concerning the role of CMV in NKG2C upregulation on γδ cells in the absence of HIV are still unclear. In kidney transplant patients, although CMV^pos^ patients had mainly NKG2A^neg^ Vδ1 cells, this was also the case in control CMV^neg^ transplant patients [91,229]; in CMV-infected newborns, increased levels of both NKG2A and C were observed on γδ T cells [98]; and in Ugandan children, a progressive increase in NKG2C+ cells was observed in infants infected by CMV during the first years of life [76]. Thus, by analogy with NK cells, it is tempting to speculate that Vδ1 expansion in HIV infection is linked to CMV infection.

The effect of CMV in HIV infection seems detrimental, as CMV-seronegative individuals recover better after ART [230]. In longitudinal studies of co-infected patients, intermittent CMV/EBV replication in blood is associated with increased HIV-DNA [231]. CMV may also contribute to the Vδ2 decline in HIV patients as Vδ2 counts correlate negatively with the reactivation of CMV, reflected by high CMV-DNA and anti-CMV antibodies [232]. However, in one study, a high NKG2C/NKG2A ratio on NK cells was a marker of slower progression of HIV disease and higher CD4 counts two years after primary HIV infection [233]. In another report [234], patients with high NKG2C on NK cells showed lower HIV-RNA at one month under ART; no CMV reactivation was detected, but all patients were seropositive for CMV and it was suggested that pre-existing CMV immunity was beneficial for recovery. Assuming that a high NKG2C/NKG2A ratio on NK cells reflects mainly CMV infection, CMV carriage would appear beneficial; this was attributed to NK cell activity in both studies, but a role of Vδ1 cells is an alternative non-exclusive possibility.

## 13. CMV and HBV/HCV

γδ T cells are enriched in liver tissue compared to peripheral blood [235,236,237], with increased proportions of Vδ2^neg^ cells. Vδ3 cells, which are almost absent in blood, represent a significant population in liver. Whereas one study reported the predominance of Vδ2 cells [236], another recent study shows that Vδ1 cells represent a major liver subset [237]. In a population of patients with chronic liver disease but no history of HBV/HCV infection, as well as healthy controls, phenotype determination and repertoire analysis by NGS showed that the liver contains a population of liver-resident CD27^lo^/CD45RA^hi^/Vδ2^neg^ cells (Vδ1^+^ or Vδ3^+^) which express the markers of tissue-resident CD69, CXCR3 and CXCR6, which are absent from blood. These cells represent liver-restricted clonotypes, and are possibly involved in liver tissue immunosurveillance [237]. A second CD27^lo^/CD45RA^lo^ population contains highly expanded clonotypes which are present in blood, and thus circulate between both compartments. Very little sharing of clonotypes between donors was observed, and most sequences were essentially private [237].

Hepatitis B and C viruses (HBV, HBC) infect approximately 250 and 70 million persons worldwide, respectively, and are known to provoke liver cirrhosis, liver failure and hepatocellular carcinoma. HBV infection is cleared in 95% of healthy adults. In contrast, 60 to 80% of HCV-infected patients progress toward a chronic liver disease (see [238,239] for recent reviews). Most available data on γδ T cells in HBV/HCV infections concern chronic infections.

An increase in blood γδ cells occurs in chronic HCV patients [83,240,241], but also in non-viral chronic liver diseases [242]. The frequency of Vδ1 cells in the liver of HCV and HCV-HIV patients correlates positively with inflammation and necrosis in liver tissue; Vδ1 cells also display an activated phenotype (CD62L^neg^CD45RO^pos^). Spectratyping of the Vδ1 TCR CDR3 regions of liver-infiltrating cells did not reveal oligoclonality [243,244]. A certain level of oligoclonality of blood γδ cells from HCV patients was revealed by NGS-based repertoire analysis, but repertoire complexity was similar in patients and controls. A more frequent expansion of Vδ3 clones was noticed in HCV patients, but their reactivity toward HCV could not be ascertained. The repertoire was also not significantly changed after treatment with direct-acting antiviral drugs [245]. These results suggest that alterations of the Vδ2^neg^ compartment may not be specifically due to HCV infection, and it has been suggested that it may rather reflect a homeostatic response to liver inflammation [244].

The Vδ2 cell compartment may also be altered in chronic HCV patients. Two studies indicate a decreased percentage and count of Vδ2 cells in blood [246,247]. The remaining cells expressed activation markers, including CD107, but were found to display a decreased expression of perforin compared to controls [246]. A reduced frequency of effector memory phenotype is observed in blood and reversed in patients with a sustained response to antiviral therapy, suggesting that differentiated effectors are recruited in tissue sites of viral replication. In vitro, ABP-activated Vδ2 cells from healthy donors displayed IFNγ-dependent antiviral activity against an HCV-infected Huh7 cell line, but the receptors involved were not determined [247].

During HBV infection, γδ T cell subsets frequencies are not dramatically altered in blood. Increased levels of both Vδ2 and Vδ2^neg^ subsets are reported in asymptomatic HBV carriers compared to healthy controls [248]. In a study by Chang et al., acute HBV infection was associated with increased Th1 markers on Vδ1 cells, whereas a chronic status was associated with increased Th1 markers on Vδ2 cells [249]. In chronic HBV infection, elevated or decreased percentages of γδ cells in blood have been reported, possibly due to the inclusion or not of patients with advanced disease [250,251] or to genetic factors [249]. A decrease in Vδ2 cells in blood during episodes of exacerbation of liver disease is also reported, with the remaining cells showing increased activation markers, increased cytolytic activity [248,250,252], high production of TNF as well as IL-17 and a variable ability to produce IFNγ upon in vitro stimulation; their ability to produce TNF, correlated with low hepatic cytolysis, suggests a role in liver tissue protection [252].

In HCV^+^ or HBV^+^ liver transplant patients, the predominance of the Vδ1 subset in blood γδ T cells has been observed in CMV^+^ but not EBV^+^ patients (on a serologic status basis) [83]. In HCV^+^ patients Vδ1 cells displayed mostly a terminally differentiated phenotype, as reported in persistent CMV infections. However, a link with CMV carriage was also seen in immunocompetent patients, in whom the presence of CMV-DNA has been examined in liver tissue by PCR. CMV infection of the liver was frequent in HBV^+^ (52%) and HCV^+^ (36%) patients. Interestingly, the presence of CMV-DNA correlated differently with liver damage in HCV^+^ or HBV^+^ livers, estimated from ALT and bilirubin levels: while CMV-DNA was associated with decreased ALT and bilirubin in the case of HBV, the reverse was observed in HCV patients, arguing for a detrimental effect of CMV in HCV infection, but not in the case of HBV [253]. Thus, CMV is likely a driver of the Vδ1 increase in HCV/HBV infections in an immunosuppressed context, and the outcome may depend on the viral co-infection context.

As with HIV infections, NK responses provide evidence that CMV modulates the responses to HBV/HCV. In co-infections, CMV seropositivity determines the expansion of highly differentiated NK cells expressing NKG2C and cytotoxic markers, and is not associated with adverse clinical outcomes, suggesting a role in limiting immunopathology [254]. 

## 14. CMV and HEV

HEV infection is usually benign and asymptomatic, and the infection is naturally cleared in immunocompetent individuals. It becomes a concern in the context of solid organ transplantation, as about 2/3 of patients progress towards chronic infection [255]. Our group has analysed the phenotype and frequency of γδ T cells in a panel of rare immunocompetent patients with acute symptomatic infections [256]; we found an elevated frequency of Vδ2^neg^ cells compared to age- and sex-matched healthy controls. Segregation of patients and controls according to CMV serology revealed that high Vδ1 frequency was associated with combined HEV infection and CMV seropositivity. Again, high Vδ1 levels were associated with a higher proportion of Vδ1 cells with a TEMRA phenotype. In vitro, γδ T cells from patients were found to upregulate CD69 activation markers when stimulated with HEV-infected hepatocarcinoma cells, and this was selective for CMV carriers. This concerned Vδ2^neg^ and Vδ2^pos^ cells, although the response of the two subsets correlated negatively. HEV infection in CMV carriers was also associated with an increased concentration of IL-10 in the serum. This indicates that CMV carriage is determinant for the expansion of Vδ2^neg^ cells, although both Vδ2^neg^ and Vδ2^pos^ subsets may be involved in the response to HEV. The data also suggest a possible role in limiting immunopathology through IL-10 production by uncharacterised cells. HEV-infected hepatocarcinoma cells also induce the upregulation of IL-10 production by co-cultivated γδ cells, suggesting alternatively that this production could provide an immune escape mechanism for HEV by dampening the activity of antiviral effectors. Strikingly, a skewing of Vδ2^pos^/Vδ2^neg^ proportions was not observed in immunosuppressed transplant patients at the acute or chronic phase [11]. Nevertheless, a depletion of more differentiated Vδ2^pos^ (CD27^−^CD45RA^−^) and Vδ2^neg^ cells (TEMRA, CD27^−^CD45RA^+^) was observed, suggesting their recruitment in tissues, as reported in the case of chronic HCV patients [247].

## 15. Is There a Role for HHVs in Other Viral Co-Infections?

The mobilisation of γδ subsets in acute viral infections in humans and animal models has been extensively reviewed, and we will discuss here the findings relevant to HHV co-infection and cross-protection conferred by different microbes [16,17,257].

Following influenza virus infection in mice (reviewed in [17]), successive waves of γδ T cell infiltration have been detected in the lung, with a first wave of pro-inflammatory Vγ4 cells followed by infiltration of Vγ2 and Vγ1.1 T cells, which are thought to control inflammation [258]. In humans, although no massive mobilisation is reported in the periphery during influenza A/B infections (IAV/IBV), IL-17-producing Vγ9Vδ2 cells are increased in the bronchial lavage fluid of infected patients, and this correlates with lower pneumonia severity [258]. Ex vivo, blood Vδ2 cells from patients produce IFNγ upon stimulation by IBV-infected cells. Responding cells present the TCR characteristics of PhAg-responsive cells and kill IBV-infected cells in a PhAg-dependent manner [61,259]. Since influenza infections are frequent and recurrent across a lifespan, they may participate in the shaping of the γδ T cell repertoire after birth [61]. Nevertheless, the reactivation of HSV, CMV and EBV is frequent in ICU patients treated for severe influenza infections, and are associated with high morbidity and mortality [260]. Influenza virus infection increases the titre of antibodies against EBV, also indicative of the reactivation of EBV, and this is also observed with the measles virus and adenovirus [261]. Thus, the interplay between these viruses and the possibility of cross-immunity/cross-reactivity deserves further investigation.

The contribution of HHV carriage is also questionable in the context of SARS-CoV and SARS-Cov-2 infections. These viruses cause an acute respiratory infection of variable severity, and Vγ9Vδ2 T cells are mobilised in both cases [262,263]. In SARS-CoV patients, an expansion of this subset was observed at a distance from the initiation of the disease (~3 months), and expanding cells were able to kill in-vitro-infected targets. In SARS-CoV-2 patients, an early depletion of the Vδ2 subset was reported, with a possible slight increase in Vδ2^neg^Vδ1^neg^ γδ cells. Remaining Vδ2 cells displayed markers of activation, suggesting activation and traffic to the lung, similar to what was observed in influenza infection in mice. Again, the reactivation of EBV in severe COVID-19 disease is a factor of morbidity and complications, but the reactivation of other HSVs can also affect the course of disease [35,264,265,266,267,268]. How this affects γδ T cells and their putative protective or deleterious activity is unknown.

## 16. HHVs in Malaria

Alterations of γδ subsets in malaria have been reviewed recently [269,270,271]. In highly endemic areas, chronic plasmodium infection leads to Vδ2 depletion in peripheral blood, while remaining cells present exhaustion markers and reduced functionality, suggestive of chronic stimulation [272,273,274,275]. Conversely, Vδ2 cells expand in primo-infected people from non-endemic areas. The highest expansion was observed at the recovery phase [276], a delay which is unexplained and may be linked to a peak of production of malarial antigens or to a role of Vδ2 cells in the resolution of infection. In vitro, Vγ9Vδ2 T cells are stimulated by Plasmodium falciparum (Pf)-infected erythrocytes, and this is due to PhAg production by parasites which carry the non-mevalonate pathway of PhAg synthesis. PhAg can be released from infected erythrocytes and stimulate Vδ2 cells after capture by other cells [277,278]. It was recently shown that infected erythrocytes express BTN3A1 and directly stimulate granulysin-dependent parasite killing by Vγ9Vδ2 T cells [279,280].

In highly endemic areas, the main observable perturbation in the γδ T cell compartment is an expansion of Vδ1 T cells displaying resting (CD69^-^DR^-^), a TEMRA phenotype (CD45RA^+^) and frequent expression of CD8. Vδ1 cells in malaria thus partly phenocopy Vδ2^neg^ γδ T cells that expand during CMV expansions [270,281,282]. Nevertheless, CDR3δ spectratyping indicated that Vδ1 expansion in chronically infected children was highly polyclonal or displayed a level of clonal focussing, similar to the one observed in healthy children from the same endemic area [281]. In a cohort of stable infected people living in highly endemic Laos, Vδ2^neg^ cells proliferated in vitro against crude Pf antigens in the presence of IL-2, produced IFNγ as well as IL-10 and were associated with high concentrations of IL-10 in plasma, suggesting anti-inflammatory potential and a role in the control of chronic inflammation. Vδ1 cells from infected donors also expanded and secreted IL-10 in the absence of Pf antigens when IL-2 was present, presumably primed, in vivo, by an unknown stimulus [283]. 

The reactivation of latent HHVs is frequent during malaria, and the reactivation of EBV is probably linked to the high incidence of Burkitt’s lymphoma in highly endemic areas [284,285,286]. An exhaustive study of the reactivation of HHV has been conducted in Ugandan children with malaria via the detection of viral DNA in plasma and saliva. All children had at least one HHV detected in saliva, most frequently HHV-6/7 (70%) and CMV (50%). The CIDR1α domain of Pf membrane protein 1 is a strong polyclonal B cell activator, possibly causing the reactivation of EBV [287]. Viral DNA was strongly diminished by antimalarial treatment, at least concerning HSV-1 or EBV, indicative of a direct link with Pf infection [288,289]. Thus, the reactivation of HHV could presumably explain Vδ1 expansions in Plasmodium infections in highly endemic areas, but this requires formal demonstration. This is not detrimental, as high Vδ1 frequencies are associated with a tolerant or stable immune status among malaria-exposed individuals [283,290,291]. 

## 17. Lessons from Mycobacterial Infections

The outgrowth of Vγ9Vδ2 T cells has been observed, in vivo, in multiple bacterial infections. Bacterial extracts from mycobacteria, but also from Gram-positive and Gram-negative bacteria, promote Vγ9Vδ2 T cell expansions. This finding led to the first identification of PhAgs from mycobacteria that produce HMBPP through the DOXP pathway [292,293]. Vγ9Vδ2 T cells provide some protection against mycobacterial and other infections, such as HMBPP-producing Listeria monocytogenes, in a simian model in particular (reviewed by Shen et al. [294]). Mycobacterium bovis BCG also produces PhAgs, leading to the expansion of Vγ9Vδ2 in vivo after infection in macaques, although this was not apparent in infants [295,296]. Vγ9Vδ2 cells can kill intracellular bacteria through a granulysin- and perforin-dependent process [297]. It seems, however, that only a subset of PhAg-reactive Vγ9Vδ2 cells are activated by BCG-infected dendritic cells [298]. Effects independent of the TCR/BTN3A axis are, however, possible, since γδ cell expansion following PhAg administration also occurred in pigs [299].

Similar to HHVs, M. tuberculosis and BCG infections lead to a state of asymptomatic latency in most individuals. Latency can last at least for 16 months and possibly for life, promoting sustained protective immunity. They can also reactivate from this state following immune depression and other favourable conditions [300,301,302].

BCG vaccination has been shown to provide protection against multiple unrelated infections, and reduces mortality in vaccinated infants far beyond its effects on tuberculosis [303]. Protection by BCG vaccination is reported toward respiratory viral infections and genital warts caused by HPV (reviewed in [304]). It reduces SARS-Cov2 infection severity [305,306,307] and improves the responses to other vaccines in humans (IAV, HBV). Remarkably, BCG vaccination is reported to reduce the reactivation of HSV infections in two clinical trials involving 15 and 109 patients, thus reducing the recurrence of genital herpes lesions [308,309]. This is in line with both infections being targeted by Vγ9Vδ2 cells, and suggests a possible protective role of memory Vγ9Vδ2 T cells through PhAg sensing. Nevertheless, this view is challenged by reports that BCG vaccination also protects mice against IAV and HSV-2 infection, in addition to multiple other viral infections. In humans, vaccination increases, in vitro, the production of IFNγ from lymphoid cells stimulated with unrelated microbial antigens such as tetanus toxoid, LPS, Candida albicans, Staphylococcus aureus or HBV [310]. Therefore, protection may also rely on unspecific mechanisms independent of Vγ9Vδ2 cells and PhAg sensing [304], or possibly on CD4 or CD8 T cells specific to non-targeted antigens [311].

## 18. Discussion and Concluding Remarks

Vγ9Vδ2 PhAg-responsive cells display innate-like properties and react and/or expand in multiple infectious contexts, including EBV, HHV1/2, HHV6, influenza and plasmodium infections. PhAg sensing is a common determinant of this activity, and raises questions about the real function of PhAgs. A straightforward hypothesis is that endogenous IPP is the primary stimulus promoting Vγ9Vδ2 expansion, helped by costimulatory pathways such as NK receptors, CD16, PRRs and innate cytokines. If this is the case, and considering persistence of memory cells, Vγ9Vδ2 would be expected to cross-react in different contexts and to confer broad cross-protection. Such a model would at least partly explain the effect of BCG, and extend the concept of cross-protection described by Barton et al. in mice infected with murine herpes viruses γHV-68 or mCMV [312], relating to conventional T cells. The model would also fit with the broad polyclonal Vγ9Vδ2 repertoire of influenza-reactive/PhAg-reactive cells [61]. Alternatively, ubiquitous IPP could serve to promote the proper expression of BTN family multimers. These possibly promote the cell surface expression of uncharacterised clonotypic ligands which could be either tissue- or pathogen-related markers of cellular stress. IPP could be viewed as a positive selection driver, possibly used also during thymic differentiation. In this case, the whole compartment would appear globally PhAg-reactive in experimental conditions (high IPP or HMBPP concentration), but individual clones would be more selective for a clonotypic ligand. This would explain why mycobacteria-reactive Vγ9Vδ2 cells represent only a subset of PhAg-reactive cells [298], but would also limit the possibility for cross-immunity.

In the human Vδ2^neg^ compartment, an adaptive-like behaviour is supported by the occurrence of massive, oligoclonal and essentially private expansions arising after birth upon encountering a pathogen. Available data suggest that a significant, although difficult to evaluate, fraction of these cells is mobilised by CMV infection; at present, there is no evidence that this virus promotes possible cross-protection or cross-immunity toward other pathogens. This is somewhat striking as no CMV antigen has been found to be a TCR ligand. One can hypothesise that this virus induces a permissive signal or determinant allowing for the recognition of stress markers recognised in a clonal fashion, as is the case for EPCR or EpHA2.

In future research it might thus be important to evaluate the cross-reactivity of γδ cells toward different pathogens which preferentially target one or the other of the Vδ2 or non-Vδ2 compartments. Indeed, there are accumulating indications that non-Vδ2 cells are mobilised selectively in CMV carriers in hepatitis B/C/E or HIV infections, but at present it is not clear if the mobilised cells effectively participate in the anti-hepatitis virus/HIV response (with a possible involvement of a permissive determinant from CMV) or if they exclusively respond to CMV. It will be important to study whether different HHVs induce or modulate alternative γδ responses and whether these are beneficial or not to the host, with the ultimate goal to determine whether HHVs should be considered symbiotic microbes.
